# Hepatic Stellate Cell-Immune Interactions in NASH

**DOI:** 10.3389/fendo.2022.867940

**Published:** 2022-06-09

**Authors:** James K. Carter, Scott L. Friedman

**Affiliations:** ^1^ Division of Liver Diseases, Icahn School of Medicine at Mount Sinai, New York, NY, United States; ^2^ Medical Scientist Training Program, Icahn School of Medicine at Mount Sinai, New York, NY, United States

**Keywords:** hepatic stellate cells, NASH, inflammation, fibrosis, immunity

## Abstract

Nonalcoholic fatty liver disease (NAFLD) is the dominant cause of liver disease worldwide. Nonalcoholic steatohepatitis (NASH), a more aggressive presentation of NAFLD, is characterized by severe hepatocellular injury, inflammation, and fibrosis. Chronic inflammation and heightened immune cell activity have emerged as hallmark features of NASH and key drivers of fibrosis through the activation of hepatic stellate cells (HSCs). Recent advances in our understanding of the molecular and cellular pathways in NASH have highlighted extensive crosstalk between HSCs and hepatic immune populations that strongly influences disease activity. Here, we review these findings, emphasizing the roles of HSCs in liver immunity and inflammation, key cell-cell interactions, and exciting areas for future investigation.

## Introduction

Nonalcoholic fatty liver disease (NAFLD) is the leading cause of liver disease globally and is projected to overtake hepatitis C as the primary indication for liver transplantation in the US and Europe, along with alcoholic liver disease (1, 2). NAFLD comprises a spectrum of liver pathology from simple steatosis with increased hepatocyte lipid content but no inflammation, termed non-alcoholic fatty liver (NAFL), to non-alcoholic steatohepatitis (NASH) characterized by hepatocyte death, inflammation, and fibrosis. NASH affects roughly 1 in 5 patients with NAFLD, conferring a sizable risk of cirrhosis and hepatocellular carcinoma (HCC) (3).

**Figure 1:**
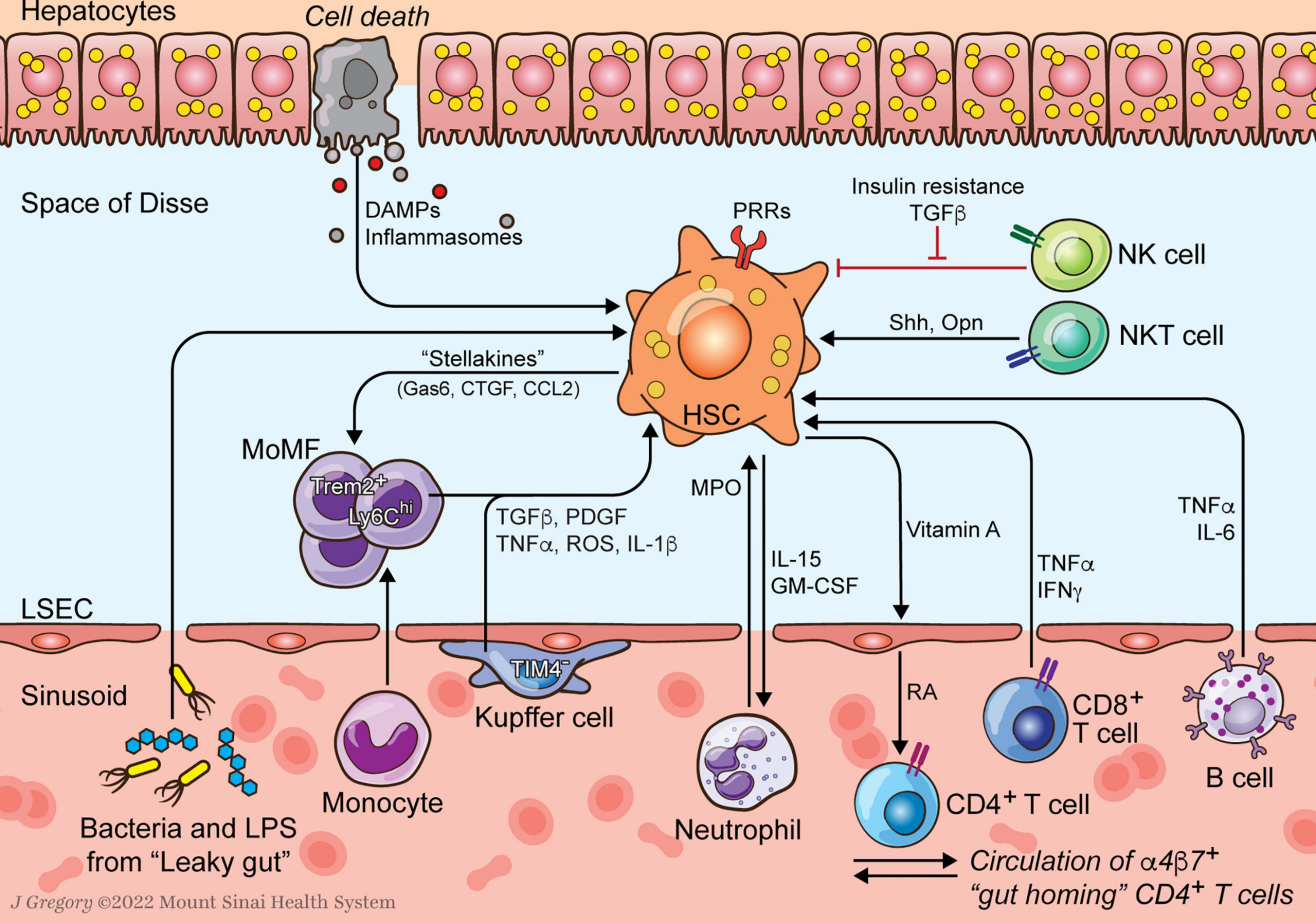
HSC-Immune Interactions in NASH Progression. Communication between hepatic stellate cells (HSCs) and immune cells amplify profibrogenic inflammatory signaling in NASH. HSCs respond to damage-associated molecular patterns (DAMPs) and pathogen-associated molecular patterns (PAMPs) that are increased in NASH. Resident Kupffer cells (KCs) and recruited monocyte-derived macrophages (MoMFs) release cytokines that promote HSC activation and survival. Neutrophil myeloperoxidase (MPO) is directly activating to HSCs which secrete factors to prolong neutrophil survival. HSCs supply vitamin A to the sinusoidal niche where it is converted to retinoic acid (RA) by liver sinusoidal endothelial cells (LSECs) as an important signal to immune cells including α_4_β_7_ integrin positive gut homing CD4^+^ T cells. Lymphocytes, including B and CD8+ T cells, release HSC-activating cytokines like TNFα and IL-6. Natural killer T (NKT) cells promote HSC activation through release of osteopontin (Opn) and sonic hedgehog (Shh). Natural killer (NK) cells display reduced HSC killing due to increased insulin and TGFβ signaling.

Sustained inflammation is thought to drive the transition from simple steatosis to NASH (4, 5). An important downstream consequence of hepatic inflammation is the activation of hepatic stellate cells (HSCs), the principal fibrogenic cell type in the liver (**Figure 1**). Fibrosis severity mirrors disease progression and is the only histologic feature that predicts liver-related mortality in NASH patients (6). Interplay between HSCs and hepatic immune cells, long recognized as a key feature of the hepatic injury response, has emerged as an especially important determinant of NASH pathogenesis (5, 7, 8). This review highlights the roles of HSCs in hepatic immunity and their impact in NASH.

## Inflammation and Immune Cell Activation in NASH

Inflammation in NAFLD is triggered by a wide range of insults that precipitate hepatocyte injury and death, and increase systemic inflammatory signals. The disease drivers in NAFLD are numerous and interdependent. Among the most significant are altered hepatocyte metabolism, lipotoxicity, and oxidative stress, which lead to endoplasmic reticulum stress and mitochondrial dysfunction. Additional dysregulation of liver homeostasis results from visceral fat inflammation, peripheral insulin resistance, and disruptions to enterohepatic bile acid circulation (3, 9). Hepatocyte-derived extracellular vesicles (EVs) carrying chemokines, inflammatory mediators, and fibrogenic micro-RNA (miR-128-3p) are increased in NASH and implicated in myeloid and HSC activation (10). Collectively, these changes lead to hepatocellular injury and increased damage- and pathogen-associated molecular patterns (DAMPs and PAMPs).

Murine models and human NASH biopsies show hepatocytes activating multiple pathways of programmed cell death. There is an increasing appreciation that lytic cell death programs, including necrosis, necroptosis, and pyroptosis are important inflammatory drivers in the pathogenesis of NALFD (11). These pathways involve rapid membrane permeabilization and release of cytoplasmic contents, triggering a stronger immune response than apoptosis (12).

Disturbance of the gut-liver axis due to NAFLD-associated dysbiosis has also emerged as an important extrahepatic source of inflammation in both experimental models and human NAFLD cohorts (13). Although disease-associated microbes vary widely between studies, the impact of changes to the composition and function of the microbiome in NASH include altered bile acid metabolism and loss of integrity of the gut-vascular barrier, precipitating leakage of LPS and bacteria into the liver *via* the portal circulation (14–16).

The liver’s rich community of innate and adaptive immune cells undergoes dramatic remodeling during the pathogenesis of NASH, with an overall increase in immune cell infiltration. Early inflammatory signals stimulate recruitment of neutrophils and accumulation of monocyte-derived macrophages (MoMFs), adding to the large liver-resident Kupffer cell (KC) population already present (17). Activated dendritic cells (DCs) increase in abundance, coordinating the adaptive immune response as professional antigen presenting cells (APCs) (18). Lymphocytes, including conventional CD4^+^ and CD8^+^ T cells, B cells, and plasma cells are educated by their interactions with APCs and adopt a range of polarized phenotypes. Similarly, multiple innate-like T cell populations are enriched in NASH in human biopsies and murine models (5).

With sustained disease, these changes to the hepatic immune composition may result in self-perpetuating, pro-inflammatory and pro-fibrogenic networks of immune cells and other liver cell types including hepatocytes, HSCs, and liver sinusoidal endothelial cells (LSECs). A major ongoing challenge is to decode how integrated networks of cells, rather than individual cell types, within the liver propagate disease pathways in NASH. Single cell RNA sequencing (scRNAseq) has granted an unprecedented view of cellular heterogeneity and transcriptional activity in the liver, and is critical to building a global understanding of the key cell-cell interactions driving disease (19–22).

## Hepatic Stellate Cells

Hepatic stellate cells (HSCs) are a versatile mesenchymal cell type with wide ranging roles in liver development, hepatocyte homeostasis, retinoid storage, and the liver’s coordinated response to injury (23). Stellate cells line the space of Disse, a niche in between sinusoidal endothelial cells and the basolateral surface of hepatocytes, establishing a central vantage point from which they monitor the hepatic environment for pathogens and hepatocellular damage. Upon liver injury, HSCs activate and transdifferentiate to generate an expanded population of myofibroblasts that are inflammatory, contractile, and produce large quantities of extracellular matrix (ECM) (24). HSC activation is an adaptive response that helps the liver respond to frequent exposure to pathogens and toxins; however, ongoing activation due to sustained liver injury leads to excess ECM deposition (ie. liver scar or hepatic fibrosis), the hallmark of chronic liver diseases including NASH (24).

HSC activation is driven by many signaling molecules and convergent pathways that conspire to initiate, and then perpetuate, the transition to a fibrogenic myofibroblast program. There are several excellent reviews of the numerous pathways involved in HSC activation including fibrogenic, proliferative, and inflammatory cytokines, Hedgehog signaling, metabolic reprogramming, cholesterol signaling, and oxidative stress (24–26). Among the most potent activating signals are cytokines and growth factors secreted by hepatic immune cells. Direct activation of HSC-expressed innate immune receptors, including the Toll-like receptors (TLRs) and complement pathway, provide additional direct activating stimuli to HSCs (8, 24). Although the primary focus thus far in studying HSC-immune crosstalk has been on its contribution to HSC activation, these interactions are bidirectional and also include contributions by HSCs as an important effector cell of the hepatic immune system. In view of the prominent role played by hepatic immune cells in NASH, HSC-immune communication is a growing area of interest (5).

## Introduction to Hepatic Stellate Cell Immune Functions

HSCs are deeply integrated into the hepatic immune system. They contribute directly to the liver’s robust and essential innate immunity through expression of a large repertoire of innate immune receptors (8, 27). In doing so they amplify pro-inflammatory cytokine production, recruit monocytes and lymphocytes to the liver, and engage directly with other effector cells of the immune system. This tight intercellular communication is ultimately reflected in the coordination between HSC activation state and hepatic immune tone (7, 8, 27, 28).

### Innate Immune Receptors

The liver is the first line of defense against invading pathogens, bacterial metabolites, and toxins entering the portal circulation from the gut. To protect against these threats, the liver recruits innate immune cells and also engages other liver cell populations including liver sinusoidal endothelial cells (LSECs), Kupffer cells (KCs), and HSCs (27).

Quiescent HSCs (qHSCs) are primed by expression of multiple pattern recognition receptors (PRRs), including TLRs 3 and 4 (29). TLR3 stimulation by double stranded RNA in qHSCs prompts IFNγ production (29). Activated HSCs (aHSCs) express an even larger set of TLRs (TLR2, TLR3, TLR4, TLR7, and TLR9) (8). The role of TLR4 and its ligand, LPS, is best characterized. When exposed to low levels of LPS, aHSCs activate the NF-κB pathway, secrete pro-inflammatory cytokines including IL-8, upregulate leukocyte adhesion molecules ICAM-1 and VCAM-1 and suppress the TGFβ pseudoreceptor Bambi (30, 31). Polymorphisms that attenuate HSC TLR4 activity reduce inflammatory signaling and are protective against fibrosis (32). TLR9 binds DNA released by apoptotic hepatocytes, stimulating fibrogenic pathways in aHSCs and halting their migration at injury sites (33).

### Immunoregulatory Functions

Beyond their roles in innate immunity, HSCs interact extensively with infiltrating immune populations and directly regulate their behavior. Activated HSCs recruit immune cells by secreting numerous chemokines including monocyte chemoattractant protein-1 (MCP-1) (34), IL-8 (35), RANTES and CCR5 (36), and SDF-1/CXCL-12 (37) and express adhesion molecules, ICAM-1 and VCAM-1, that promote the infiltration of leukocytes and macrophages (38, 39). HSCs can acquire some features of antigen presenting cells (APCs), including expression of major histocompatibility complexes (MHC) class I and II, and T cell-activating costimulatory molecules such as CD86 (40–42), although more recent evidence suggests HSCs have limited APC capabilities *in vivo* (43).

Surprisingly, several mechanisms have been identified by which activated HSCs are capable of promoting a tolerogenic environment in the liver. In co-culture experiments, HSCs interfere with T cell priming by dendritic cells (DCs) *via* a CD54 (ICAM-1)-dependent signaling pathway and by inducing STAT3 signaling in DCs (44, 45). HSCs cull the pool of activated T cells by expressing PD-L1 to induce T cell apoptosis (46–48) Remarkably, HSCs may oppose B cell activity *via* the same mechanism (49). They also amplify populations of tolerizing immune cells including FoxP3^+^ regulatory T cells (via retinoic acid and TGFβ signaling) (41, 43, 50, 51) and myeloid-derived suppressor cells (52). Supporting these physiologic roles in promoting hepatic immune tolerance are studies in which HSCs are deleted, leading to increases in the numbers of CD4^+^, CD8^+^ T cells, DCs, natural killer (NK) cells, regulatory T cells, and Ly-6C^+^ macrophages (53). The practical impact of these proposed tolerogenic roles for aHSC, which would appear to conflict with their inflammatory properties, remains uncertain. In chronic liver disease, the balance of HSC-immune interaction favors inflammatory, pro-fibrogenic signaling; however, it is possible that a minority population of tolerogenic HSCs may be identified in future studies.

### HSC – Immune Cell Interactions

From their perch along fenestrated LSECs in the perisinusoidal space, HSCs communicate directly with both resident liver cell populations as well as infiltrating immune cells. Recent single cell RNA sequencing data from the liver have reinforced the HSC’s role as a key signaling hub with many immune cell communications. Algorithms that analyze ligand-receptor pairs to predict cell-cell signaling have demonstrated that HSCs have among the greatest number and most diverse interactions of any hepatic cell type including with KCs, MoMF and to a lesser extent T, B, and NKT cells (19, 54). The number of predicted HSC-cell interactions is greatly increased upon HSC activation, highlighting the importance of these contacts during disease progression (54).

HSC-immune crosstalk is most clearly illustrated by interactions with macrophages. Upon liver injury, signaling between HSCs and macrophages coordinates the activation of both populations. Macrophages are the main cellular source of potent pro-fibrogenic signals including TGFβ, PDGF, TNF, and IL-1β, and also secrete matrix metalloproteinases (MMPs) that activate latent TGFβ stored in the ECM (24, 55). ScRNAseq of human cirrhotic livers has identified a subset of TREM2^+^CD9^+^ macrophages enriched in heavily scarred regions of the liver that secrete cytokines and growth factors, including epidermal growth factor (EGF) and platelet-derived growth factor BB (PDGF-BB) to create a pro-fibrogenic niche for aHSCs (56). HSCs, in a reciprocal fashion, can amplify the fibrogenic activity of macrophages. In a co-culture system aHSCs induced a pro-inflammatory profile in macrophages through a p38-dependent signaling pathway (57). Even so, parallel lines of communication between HSCs and macrophages appear to limit the extent of liver damage. HSCs release signals such as CX3CL1 that restrain pro-fibrogenic signaling by macrophages even during ongoing injury (58, 59). Moreover, infusion of bone marrow-derived macrophages in mice reduces fibrogenesis *via* increased matrix-degrading MMPs and anti-inflammatory IL-10 (60). These effects are likely due to the presence of “restorative macrophages” characterized by low expression of Ly-6C (in mice), elevated matrix remodeling enzymes and phagocytosis of apoptotic bodies (61, 62). The delicate balance between HSCs and macrophages has recently been distilled into a model of paracrine signaling between the two cell types that produces a stable two-cell circuit which largely predicts their behaviors under healthy and disease conditions (63, 64).

Interactions between HSCs and other immune populations are less extensively catalogued but important. HSC activation in experimental fibrosis models is reduced in immunodeficient (SCID) mice and rescued by adoptive transfer of lymphocytes, especially CD8^+^ T cells (65). Similarly, B cells and HSCs form a pro-fibrogenic network in mice subjected to CCl_4_ liver injury. Retinoic acid (RA) signaling by HSCs promotes B cell survival and activation while, in turn, B cells secrete inflammatory cytokines (66). Retinoic acid signaling has emerged as an important immunomodulatory mediator, particularly in promoting Th17 cell differentiation. RA receptor (RAR) synthetic agonists and all-trans retinoic acid (ATRA) have also shown direct anti-fibrotic effects on HSCs (67, 68).

In other contexts, immune cells may reign in HSC fibrogenic responses. Neutrophils, signaling to macrophages, facilitate the switch from disease progression to resolution – a phase when liver injury has ceased and there is a global shift away from inflammatory signaling and toward repair, including fibrosis regression (69). Similarly, interferon-γ (IFNγ), produced by many immune cells including NK, NKT and T cells, has direct anti-fibrogenic activity on HSCs (70–72).

## HSCs and Immunity in NASH

The ascendance of NASH as a worldwide health concern has spurred investigations into the immunologic pathways responsible for disease progression. These efforts have been aided by the development of murine models that capture many of the key histologic and transcriptomic features of human NASH, and by the application of single cell RNA sequencing to the liver (24, 73). The sections below highlight recent advances and summarize the pathways by which HSCs communicate with the immune system in NASH.

### Innate Immunity

Innate immune activation is an important fibrogenic stimulus in NASH (3). While it is beyond the scope of this work to provide a comprehensive review of innate immunity in NASH [see (9, 74)], the prominent contributions of inflammasome activation and changes to the microbiome are highlighted here (**Figure 1**).

Inflammasomes are multiprotein innate immune receptors present in the cytoplasm of hepatocytes and nonparenchymal liver cells. Upon stimulation by hepatocellular injury or pathogens, inflammasomes activate caspase-1 triggering release of inflammatory cytokines IL-1β and IL-18 and programmed cell death (9). The NLRP3 inflammasome has emerged as a key mediator of the transition from steatosis to NASH. In addition to the propagation of hepatic inflammation, the NLRP3 inflammasome can activate HSCs directly. Selective expression of a constitutively active transgenic NLRP3 in mouse HSCs is sufficient to induce fibrosis (75). Additionally, activated NLRP3 particles released by hepatocytes undergoing pyroptosis are endocytosed by HSCs, triggering activation and increased IL-1β production (76). Blockade of NLRP3 signaling with a small molecule inhibitor reduces disease severity in the MCD – Foz/Foz murine NASH model, although the relative contributions of targeting other inflammasome-expressing populations, including myeloid cells, have not been determined (77).

NAFLD-related changes to the composition of the gut microbiome and impaired intestinal barrier function flood the liver with PAMPs including the potent TLR4 ligand, LPS (78). These changes are positively correlated with NASH severity and fibrosis in patient cohorts and experimental NASH models (16, 79–82). Restoration of gut barrier integrity in a high fat diet (HFD) mouse model of NASH dampens innate immune signaling and reduces HSC activation (16).

#### Macrophages

Macrophages in the healthy liver are made up of KCs and circulating MoMFs. KCs maintain normal liver homeostasis as an important phagocytic cell type and essential component of the sinusoidal niche, but are stimulated by injury signals in NAFLD, prompting them to adopt an activated expression profile and recruit large numbers of inflammatory Ly-6C^hi^ MoMFs. This inflammatory switch is one of the definitive steps in the transition from NAFLD to NASH and the progression of fibrosis (4). Sustained disease drives a shift towards “alternatively activated M2-type” macrophages, characterized by heightened fibrogenic signaling (83).

Macrophages respond to a unique cocktail of injury signals in NASH, including signs of hepatocyte injury, cholesterol and lipid metabolites, and LPS from the “leaky gut” caused by NAFLD-associated dysbiosis (84). Circulating mitochondrial DNA is a powerful inflammatory signal in NASH that activates the antiviral response molecule, STimulator of Interferon Genes (STING), in macrophages (85, 86). In response to these stimuli, macrophages secrete cytokines, chemokines, and other soluble signals that contribute to fibrosis by: (A) driving HSC activation through release of TGFβ, PDGF, TNF, FGF2, MCP1, CCL3, CCL5 and reactive oxygen species (ROS), and; (B) promoting aHSC survival by activating the NFκB pathway with IL-1 and TNF (24). Not surprisingly, depleting hepatic macrophages in mouse models with agents such as clodronate liposomes, or blocking their recruitment pharmacologically, as with the CCR2/CCR5 antagonist Cenicriviroc, attenuate fibrosis and blunt other histologic markers of disease (87–91). PPARδ agonism can modulate MoMF gene expression to improve lipid handling and decrease pro-fibrogenic signaling to HSCs, synergizing with pan-PPAR agonism as a potential NASH therapy (92).

Recent studies have revealed how the hepatic macrophage population changes in response to NASH, with implications for their interactions with HSCs. A TREM2^+^ macrophage subtype that is strongly linked to markers of tissue injury and fibrosis is enriched in NASH livers (19). The role of TREM2^+^ macrophages in NASH is unsettled, and recent evidence suggests they may contribute to an adaptive response to metabolic injury in NASH and promote fibrosis regression (93, 94).

The pool of resident KCs undergoes maladaptive changes in NASH. This was highlighted by two recent studies that used mouse models of NASH to explore changes to hepatic macrophages during disease progression (20, 95). Using a combination of KC-specific markers, parabiosis studies, and bone marrow transplant experiments, they demonstrated that liver resident TIM4^+^ KCs die and are replaced by a KC-like population derived from Ly-6C^hi^ MoMFs that are more inflammatory than their predecessors. Intriguingly, HSCs, along with LSECs and hepatocytes, provide niche cues that recruit MoMFs and instruct their differentiation to the KCs fate (96). This finding raises the prospect that HSCs contribute to the acquisition of a more inflammatory and pro-fibrogenic KC population in NASH. Those MoMFs not fated to become KCs remain in the liver as CLEC4F^-^ SPP1^+^ TREM2^+^ CD9^+^ “Lipid-Associated Macrophages” (LAMs) with more activated transcriptional profiles. This population was found to associate closely with aHSCs and likely overlaps with the scar-associated TREM2^+^ CD9^+^ macrophages described by Ramachandran et al. in scRNAseq of human cirrhosis (20, 56). Finally, studies of NASH histology have identified rings of macrophages forming “Crown-like Structures” (hCLS) around dying hepatocytes that were initially thought to represent a more inflammatory macrophage subset (97, 98). Interestingly, reduced hCLS formation in Ccr2 KO mice fed a HFD was associated with similar weight gain and steatosis, but increased fibrosis, suggesting a protective role for macrophages in this context (99).

Extensive cell-cell communications between HSCs and macrophages in NASH is an area of exciting new investigation. Analysis of receptor ligand pairs using scRNAseq from the AMLN murine NASH model identified a set of HSC-specific secreted factors termed “stellakines” and established HSCs as a signaling hub that interacts extensively with LSECs, macrophages, and to a lesser extent, DCs, T cells, and B cells. The immune-targeting “stellakines”, which are upregulated in NASH, included CCL2, CCL11, CXCL10, CXCL12, CXCL16, CTGF, and Gas6 (19).

Further evidence for HSC-macrophage crosstalk emerged in a characterization of the MerTK receptor. MerTK is a surface receptor predominantly expressed by macrophages that binds several ligands including the “stellakine” Gas6. When activated it induces TGFβ production. ADAM17 cleaves MerTK to control macrophage inflammatory responses in steatotic livers, but this compensation fails in NASH – possibly due to reduced availability of HSC-supplied vitamin A (which is depleted when HSCs activate) that is necessary to stimulate ADAM17 (100).

While early studies of macrophage depletion in the CCl_4_ mouse model of liver fibrosis characterized a Ly-6C^lo^ restorative macrophage subset, additional studies are needed to clarify how macrophages specifically contribute to disease resolution in NASH (61). Emerging evidence points to Specialized Proresolving Mediators (SPMs) including maresins and resolvins as important players. At least some of these lipid metabolites are produced by macrophages (101) and also signal to macrophages to reduce inflammatory and fibrogenic gene expression (102). Other pathways to limit macrophage-HSC fibrogenic signaling are likely awaiting discovery. For example, investigators used a novel metabolomic and stable isotope tracing approach to uncover a hepatocyte-macrophage acetoacetate exchange that blocks fibrogenic signaling to HSCs in a HFD mouse model, although the detailed HSC-macrophage signaling pathways remain uncertain (103).

#### Neutrophils

**Figure 2:**
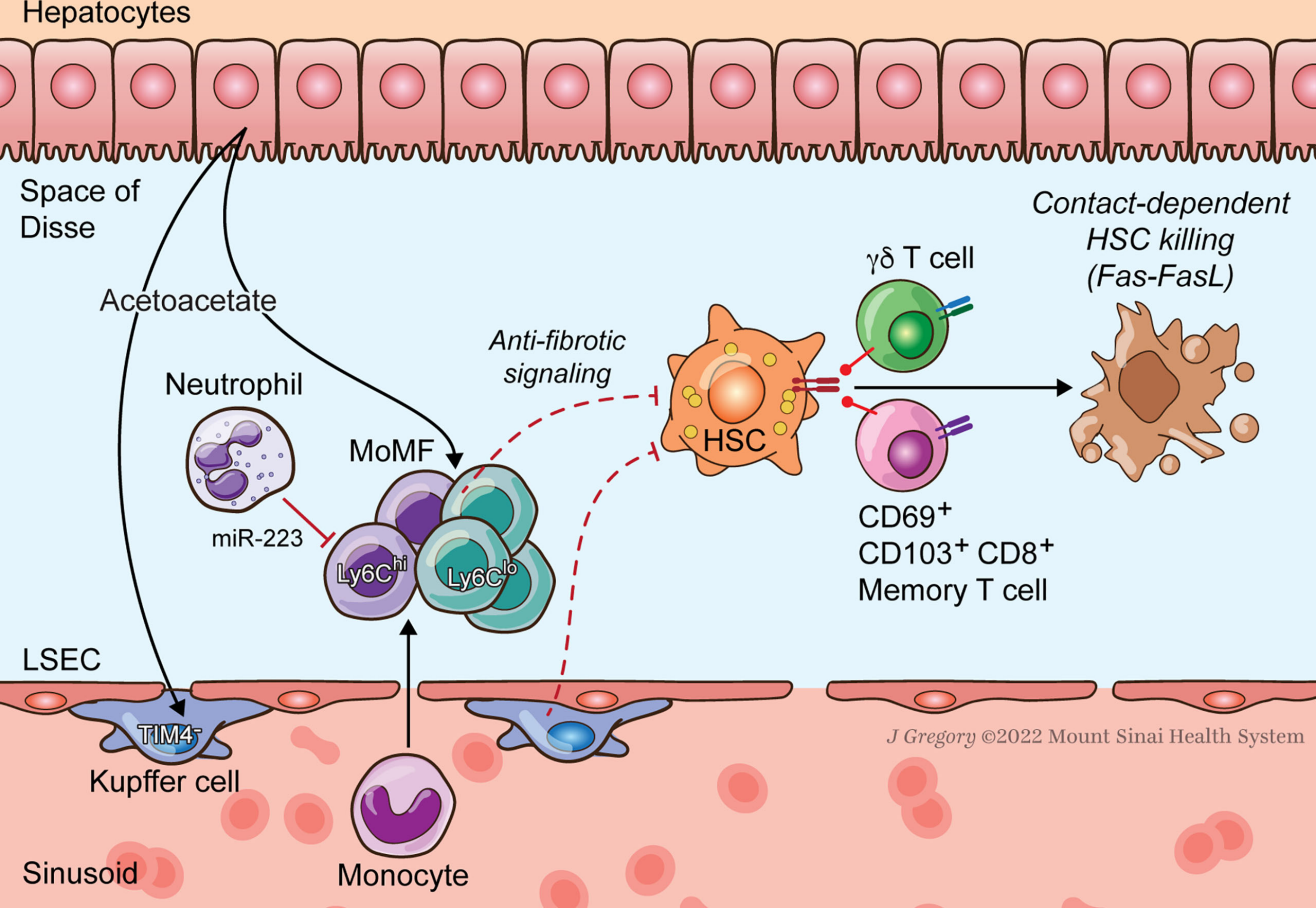
HSC-Immune Interactions in NASH Regression. Key immune pathways oppose fibrogenesis during resolution of NASH injury. CD8^+^ memory and γδT cells induce apoptosis of activated HSCs. Neutrophil microRNA 223 (miR-223) converts pro-inflammatory macrophages to a restorative phenotype. Hepatocyte-released acetoacetate promotes anti-fibrogenic signaling by macrophages.

As first responders in innate immunity, neutrophils amplify early inflammatory insults in NAFLD. Accordingly, depleting or impairing neutrophils is protective in experimental NASH models (104). HSCs are primarily activated downstream of liver injury mediated by inflammatory and oxidative neutrophil effector functions. Release of myeloperoxidase, an enzyme that catalyzes the production of multiple oxidant species, is particularly harmful in the MCD NASH model. In humans, hepatic MPO content is positively correlated with NASH severity (105, 106). MPO is also directly stimulatory towards cultured HSCs and can activate latent TGFβ in liver homogenates, pointing to pathways of direct HSC activation by neutrophils (105). During inflammatory injury, HSCs may form a positive feedback loop with neutrophils by secreting factors such as GM-CSF and IL-15 that extend the neutrophil half-life, at least in co-culture experiments (107). As observed with other immune cell types, neutrophils may have dual functions in NASH depending on disease stage. Depletion of neutrophils after establishment of liver injury and during the resolution phase of disease *impairs* fibrosis regression in the MCD NASH model, where neutrophils may be an essential source of miR-223, a microRNA that suppresses the NLRP3 inflammasome in macrophages (69) (**Figure 2**).

#### NK Cells

Early studies in experimental liver fibrosis indicated that NK cells were programmed to selectively target and kill activated HSCs, raising hopes that NK cells could have fibrolytic activity in chronic liver diseases (108–111). Activated NK cells also secrete antifibrotic IFNγ, although in advanced fibrosis HSCs inhibit NK cells through release of TGF-β (Jeong Hepatology 2011). In NASH, increased recruitment of NK cells to the liver is correlated with more severe disease (112). Part of the reason NK cells fail to control fibrogenesis in NASH may be that their HSC-targeting activity is being inhibited. NK cells isolated from a cohort of patients with NAFLD-NASH displayed abrogated HSC killing when derived from individuals with more severe insulin resistance and advanced fibrosis (113). In a murine NAFLD model, increased TGFβ signaling led to loss of NK cell cytolytic activity – potentially an adaptive mechanism to reduce liver inflammation (114). Together, these studies suggest that worsening insulin resistance and fibrogenic TGFβ signaling during NASH progression may blunt anti-myofibroblast surveillance by NK cells.

#### NKT Cells

NKT cells are the main unconventional innate-like T cells in the liver, along with γδT cells and mucosal-associated invariant T (MAIT) cells (115). NKT cells have emerged as key drivers of hepatocyte injury during NASH pathogenesis, but the significance of direct NKT-HSC interactions is not well characterized (115, 116).

NKT cells secrete HSC activating ligands sonic hedgehog (Shh) and osteopontin (Opn) in the MCD mouse model of NASH (117–120). Although other hepatic cell types produce Opn, NKT-deficient Jα18^-/-^ and CD1d^-/-^ transgenic mice have lower total hepatic Opn levels and reduced fibrosis. Moreover, NKT conditioned medium stimulates HSC activation in culture, suggesting a direct fibrogenic role for NKT paracrine signaling (118). In a co-culture system, NKT cells from the CD-HFD NASH model activate HSCs more strongly than CD8^+^ T cells, even though both immune populations mediate fibrogenic injury *in vivo* (121). Intriguingly, studies in CCl_4_ and DDC liver fibrosis models have reported that NKT cells can kill activated HSCs through expression of the NK receptor NKG2D, but its relevance to NASH has not been explored (122, 123).

#### Other Innate-Like T cells

Hepatic γδT cells are highly enriched in both healthy and diseased livers (124). Upon exposure to activated HSCs γδT cells induce apoptosis through the Fas-FasL pathway and depleting the liver of γδT cells in CCR6^-/-^ mice accelerates MCD-NASH-related fibrosis (125). Like NKT cells, γδT cells appear to upregulate NK receptors to acquire HSC-targeting capacity under injury conditions (126). MAIT cells have recently emerged as a source of profibrogenic signaling in chronic liver diseases, but their contribution in NASH has not been clarified (127, 128).

### Adaptive Immunity

Adaptive immune responses are potent inflammatory drivers in the progression to NASH. Hepatocyte and LSEC-derived signals recruit a broad repertoire of lymphocytes, resulting in the diffuse lobular infiltration that is a hallmark of NASH histology (116, 129). Global loss of adaptive immunity in Rag1^-/-^ and β2M^-/-^ mice is protective against steatosis, inflammation, and fibrosis in the choline-deficient high fat diet (CD-HFD) model, highlighting the overall contribution of adaptive immune cells to NASH disease (121).

#### Conventional T Cells

T cells may be especially important in promoting NASH and NASH-HCC (116, 130). The NASH T cell response is characterized by an enrichment for cytotoxic CD8^+^ T, T_H_1 differentiated CD4^+^ T and NKT cell populations (18, 121, 131–135). These immune shifts drive hepatocyte metabolic dysregulation through IFNγ, TNFα, and IL-17A signaling and production of the lymphotoxin LIGHT (18, 121, 132, 133). Metabolic derangement in NASH also induces T cell pathology. Notably, CXCR6^+^ CD8^+^ T cells acquire hepatocyte killing activity in response to increased levels of the short-chain fatty acid acetate (136). Accordingly, depletion of T lymphocyte populations or disruption of their signaling activity is protective (115, 133, 137).

HSCs are an integral part of the sinusoidal niche and likely provide signals that influence T cell functions in NASH. Receptor-ligand analysis of their dense signaling networks includes potential chemokine interactions with T cells (19). Moreover, HSCs are the key hepatic source of vitamin A and its immunologically important metabolite RA (8). Vitamin A from HSCs is converted by neighboring LSECs to RA which primes CD4^+^ T cells to acquire a gut-homing phenotype mediated by α_4_β_7_ integrin and CCR9 expression (42, 138). These α4β7^+^ CD4^+^ T cells are key mediators of intestinal barrier disruption, increasing enterohepatic circulation of LPS and bacteria. Antagonism of this pathway in an experimental mouse model of NASH reduces hepatic inflammation and fibrosis (139).

T cells may act to restrain HSC fibrogenesis during NASH regression (**Figure 2**). In a HFD NASH model there was expansion of CD69^+^ CD103^−^ CD8^+^ tissue resident memory T cells when mice were allowed to recover on a regular chow diet. These CD8^+^ memory T cells attract HSCs through the chemokine receptor CCR5 and induce HSC apoptosis *via* Fas-FasL. Adoptive transfer of regression-primed CD8^+^ memory T cells reduces the number of aHSCs and controls fibrosis (21).

#### B Cells

Intrahepatic B cell infiltration is associated with NASH progression (140, 141). Soluble markers related to B cell survival and activity including BAFF and IgG increase with worsening NASH severity, but the mechanisms by which B cells contribute to disease have not been characterized (142, 143). In HFD-NAFLD and MCD-NASH mouse models, B cells display prominent innate-like signaling function characterized by release of pro-inflammatory TNFα and other cytokines that activate HSCs and promote T_H_1 T cell activity (140, 144, 145). Future studies empowered by advances in scRNAseq and associated technologies may identify additional points of communication between B cells and HSCs (145). Potential interactions are hinted at, but not explored, in some of the first single cell analyses of NASH livers, including Cxcl12-Ccr4 in mouse and TNFRSF14-BTLA in human aHSCs and B cells, respectively (19, 146).

## Emerging Areas

Cellular heterogeneity is now evident among HSCs and immune cell subsets as a result of increasing use of scRNAseq, raising new questions about how interactions between these cell types may be subdivided and targeted with greater precision (19, 20, 145, 147). At the same time, there is growing appreciation for antifibrotic signaling by immune cells, especially during fibrosis regression. Future studies that can identify pro-fibrotic and antifibrotic cell types and interactions with greater precision will be of great value to the development of targeted therapies.

Single cell analysis of HSC populations in murine NASH recently identified a cluster of “inflammatory” HSCs characterized by reduced collagen scar production and increased immune and secretory pathway activity that may be more relevant to immune crosstalk (148). Separately, fibrogenic TREM2^+^CD9^+^ “scar-associated” macrophages have been characterized in human cirrhosis (56). These may represent key pathologic cellular subsets worthy of therapeutic targeting. Likewise, depletion of other disease-associated subpopulations such as senescent HSCs, which are inflammatory, immune-stimulating in NASH, would be an appealing approach; however, these studies will rely on characterizing a unique molecular signature of HSCs to selectively target this cell type (149, 150). Any cell-directed therapies will need to avoid inhibition of beneficial activities by cells including “restorative” Ly-6C^lo^ macrophages and miR-223-producing neutrophils. In support of this, additional work is needed to further define the cell subsets with antifibrotic roles, especially those that promote fibrosis regression.

## Conclusions

Interactions between HSCs and hepatic immune cells clearly regulate fibrosis in NASH. HSCs collaborate with innate immune cell types to initiate hepatic inflammation in the transition from simple steatosis to steatohepatitis. They also undergo fibrogenic activation in response to inflammatory signaling, mediated by both innate and adaptive cell types, and signal back to those immune subsets, amplifying their activation (**Figure 1**) (3, 9). However, increasing evidence indicates that immune-HSC crosstalk is also tightly linked in the resolution of NASH injury and fibrosis regression. In specific contexts NK, NKT, and CD8^+^ T cells all induce HSC apoptosis to attenuate scar deposition (21, 108, 123). Neutrophil and macrophage subsets may blunt HSC activation and promote fibrosis regression as well (**Figure 2**) (69, 103).

Future studies need to establish the key signals that orchestrate the shift from pro- to antifibrotic signaling by immune cells. Many new candidate interactions are already suggested through single cell transcriptomic analyses, enabling a global assessment of immune cell interaction networks (147, 151, 152) Ultimately, functional validation will be necessary to establish which interactions are most impactful.

Careful dissection of timing and key regulators of HSC-immune interactions in NASH promises to clarify which therapeutic strategies will disrupt disease promoting pathways without interfering with the liver’s innate capacity for repair.

## Author Contributions

JC and SF wrote the manuscript. Both authors contributed to the article and approved the submitted version.

## Funding

SF is supported by the National Institutes of Health (NIH) R01DK128289-01. JC receives support from NIH T32 GM007280.

## Conflict of Interest

The authors declare that the research was conducted in the absence of any commercial or financial relationships that could be construed as a potential conflict of interest.

## Publisher’s Note

All claims expressed in this article are solely those of the authors and do not necessarily represent those of their affiliated organizations, or those of the publisher, the editors and the reviewers. Any product that may be evaluated in this article, or claim that may be made by its manufacturer, is not guaranteed or endorsed by the publisher.

## References

[1] Wong RJ Aguilar M Cheung R Perumpail RB Harrison SA Younossi ZM. Nonalcoholic Steatohepatitis is the Second Leading Etiology of Liver Disease Among Adults Awaiting Liver Transplantation in the United States. Gastroenterology (2015) 148(3):547–55. doi: 10.1053/j.gastro.2014.11.039 25461851

[2] Younossi ZM. Non-Alcoholic Fatty Liver Disease - A Global Public Health Perspective. J Hepatol (2019) 70(3):531–44. doi: 10.1016/j.jhep.2018.10.033 30414863

[3] Loomba R Friedman SL Shulman GI. Mechanisms and Disease Consequences of Nonalcoholic Fatty Liver Disease. Cell (2021) 184(10):2537–64. doi: 10.1016/j.cell.2021.04.015 PMC12168897 33989548

[4] Krenkel O Tacke F. Liver Macrophages in Tissue Homeostasis and Disease. Nat Rev Immunol (2017) 17(5):306–21. doi: 10.1038/nri.2017.11 28317925

[5] Huby T Gautier EL. Immune Cell-Mediated Features of non-Alcoholic Steatohepatitis. Nat Rev Immunol (2021) 5:1–15. doi: 10.1038/s41577-021-00639-3 PMC8570243 34741169

[6] Hagstrom H Nasr P Ekstedt M Hammar U Stal P Hultcrantz R. Fibrosis Stage But Not NASH Predicts Mortality and Time to Development of Severe Liver Disease in Biopsy-Proven NAFLD. J Hepatol (2017) 67(6):1265–73. doi: 10.1016/j.jhep.2017.07.027 28803953

[7] Maher JJ. Interactions Between Hepatic Stellate Cells and the Immune System. Semin Liver Dis (2001) 21(3):417–26. doi: 10.1055/s-2001-17555 11586469

[8] Weiskirchen R Tacke F. Cellular and Molecular Functions of Hepatic Stellate Cells in Inflammatory Responses and Liver Immunology. Hepatobiliary Surg Nutr (2014) 3(6):344–63. 10.3978/j.issn.2304-3881.2014.11.03 PMC4273119 25568859

[9] Schuster S Cabrera D Arrese M Feldstein AE. Triggering and Resolution of Inflammation in NASH. Nat Rev Gastroenterol Hepatol (2018) 15(6):349–64. doi: 10.1038/s41575-018-0009-6 29740166

[10] Hirsova P Ibrahim SH Verma VK Morton LA Shah VH LaRusso NF. Extracellular Vesicles in Liver Pathobiology: Small Particles With Big Impact. Hepatology (2016) 64(6):2219–33. doi: 10.1002/hep.28814 PMC5115968 27628960

[11] Beier JI Banales JM. Pyroptosis: An Inflammatory Link Between NAFLD and NASH With Potential Therapeutic Implications. J Hepatol (2018) 68(4):643–5. doi: 10.1016/j.jhep.2018.01.017 PMC6185810 29408544

[12] Gautheron J Gores GJ Rodrigues CMP. Lytic Cell Death in Metabolic Liver Disease. J Hepatol (2020) 73(2):394–408. doi: 10.1016/j.jhep.2020.04.001 32298766 PMC7371520

[13] Brandl K Schnabl B. Intestinal Microbiota and Nonalcoholic Steatohepatitis. Curr Opin Gastroenterol (2017) 33(3):128–33. doi: 10.1097/MOG.0000000000000349 PMC5662009 28257306

[14] Schnabl B Brenner DA. Interactions Between the Intestinal Microbiome and Liver Diseases. Gastroenterology (2014) 146(6):1513–24. doi: 10.1053/j.gastro.2014.01.020 PMC3996054 24440671

[15] Sydor S Best J Messerschmidt I Manka P Vilchez-Vargas R Brodesser S. Altered Microbiota Diversity and Bile Acid Signaling in Cirrhotic and Noncirrhotic NASH-HCC. Clin Transl Gastroenterol (2020) 11(3):e00131. doi: 10.14309/ctg.0000000000000131 32352707 PMC7145043

[16] Mouries J Brescia P Silvestri A Spadoni I Sorribas M Wiest R. Microbiota-Driven Gut Vascular Barrier Disruption is a Prerequisite for Non-Alcoholic Steatohepatitis Development. J Hepatol (2019) 71(6):1216–28. doi: 10.1016/j.jhep.2019.08.005 PMC6880766 31419514

[17] Feng D. The Alteration of Immune Cells in the Pathogenesis of non-Alcoholic Fatty Liver Disease and non-Alcoholic Steatohepatitis. Liver Res (2020) 4(1):23–7. doi: 10.1016/j.livres.2020.02.003

[18] Henning JR Graffeo CS Rehman A Fallon NC Zambirinis CP Ochi A. Dendritic Cells Limit Fibroinflammatory Injury in Nonalcoholic Steatohepatitis in Mice. Hepatology (2013) 58(2):589–602. doi: 10.1002/hep.26267 23322710 PMC3638069

[19] Xiong X Kuang H Ansari S Liu T Gong J Wang S. Landscape of Intercellular Crosstalk in Healthy and NASH Liver Revealed by Single-Cell Secretome Gene Analysis. Mol Cell (2019) 75(3):644–60.e645. doi: 10.1016/j.molcel.2019.07.028 31398325 PMC7262680

[20] Remmerie A Martens L Thone T Castoldi A Seurinck R Pavie B. Osteopontin Expression Identifies a Subset of Recruited Macrophages Distinct From Kupffer Cells in the Fatty Liver. Immunity (2020) 53(3):641–57.e614. doi: 10.1016/j.immuni.2020.08.004 32888418 PMC7501731

[21] Koda Y Teratani T Chu PS Hagihara Y Mikami Y Harada Y. CD8(+) Tissue-Resident Memory T Cells Promote Liver Fibrosis Resolution by Inducing Apoptosis of Hepatic Stellate Cells. Nat Commun (2021) 12(1):4474. doi: 10.1038/s41467-021-24734-0 34294714 PMC8298513

[22] Deczkowska A David E Ramadori P Pfister D Safran M Li B. XCR1(+) Type 1 Conventional Dendritic Cells Drive Liver Pathology in non-Alcoholic Steatohepatitis. Nat Med (2021) 27(6):1043–54. doi: 10.1038/s41591-021-01344-3 34017133

[23] Friedman SL. Hepatic Stellate Cells: Protean, Multifunctional, and Enigmatic Cells of the Liver. Physiol Rev (2008) 88(1):125–72. doi: 10.1152/physrev.00013.2007 PMC2888531 18195085

[24] Tsuchida T Friedman SL. Mechanisms of Hepatic Stellate Cell Activation. Nat Rev Gastroenterol Hepatol (2017) 14(7):397–411. doi: 10.1038/nrgastro.2017.38 28487545

[25] Lee YA Wallace MC Friedman SL. Pathobiology of Liver Fibrosis: A Translational Success Story. Gut (2015) 64(5):830–41. doi: 10.1136/gutjnl-2014-306842 PMC4477794 25681399

[26] Schwabe RF Tabas I Pajvani UB. Mechanisms of Fibrosis Development in Nonalcoholic Steatohepatitis. Gastroenterology (2020) 158(7):1913–28. doi: 10.1053/j.gastro.2019.11.311 PMC7682538 32044315

[27] Gao B Jeong WI Tian Z. Liver: An Organ With Predominant Innate Immunity. Hepatology (2008) 47(2):729–36. doi: 10.1002/hep.22034 18167066

[28] Gupta G Khadem F Uzonna JE. Role of Hepatic Stellate Cell (HSC)-Derived Cytokines in Hepatic Inflammation and Immunity. Cytokine (2019) 124:154542. doi: 10.1016/j.cyto.2018.09.004 30241896

[29] Wilson CL Mann J Walsh M Perrugoria MJ Oakley F Wright MC. Quiescent Hepatic Stellate Cells Functionally Contribute to the Hepatic Innate Immune Response via TLR3. PloS One (2014) 9(1):e83391. doi: 10.1371/journal.pone.0083391 24416163 PMC3885413

[30] Paik YH Schwabe RF Bataller R Russo MP Jobin C Brenner DA. Toll-Like Receptor 4 Mediates Inflammatory Signaling by Bacterial Lipopolysaccharide in Human Hepatic Stellate Cells. Hepatology (2003) 37(5):1043–55. doi: 10.1053/jhep.2003.50182 12717385

[31] Seki E De Minicis S Osterreicher CH Kluwe J Osawa Y Brenner DA. TLR4 Enhances TGF-Beta Signaling and Hepatic Fibrosis. Nat Med (2007) 13(11):1324–32. doi: 10.1038/nm1663 17952090

[32] Guo J Loke J Zheng F Hong F Yea S Fukata M. Functional Linkage of Cirrhosis-Predictive Single Nucleotide Polymorphisms of Toll-Like Receptor 4 to Hepatic Stellate Cell Responses. Hepatology (2009) 49(3):960–8. doi: 10.1002/hep.22697 PMC2891538 19085953

[33] Watanabe A Hashmi A Gomes DA Town T Badou A Flavell RA. Apoptotic Hepatocyte DNA Inhibits Hepatic Stellate Cell Chemotaxis via Toll-Like Receptor 9. Hepatology (2007) 46(5):1509–18. doi: 10.1002/hep.21867 17705260

[34] Marra F Valente AJ Pinzani M Abboud HE. Cultured Human Liver Fat-Storing Cells Produce Monocyte Chemotactic Protein-1. Regulation by Proinflammatory Cytokines. J Clin Invest (1993) 92(4):1674–80. 10.1172/JCI116753 PMC288326 8408620

[35] Schwabe RF Schnabl B Kweon YO Brenner DA. CD40 Activates NF-Kappa B and C-Jun N-Terminal Kinase and Enhances Chemokine Secretion on Activated Human Hepatic Stellate Cells. J Immunol (2001) 166(11):6812–9. doi: 10.4049/jimmunol.166.11.6812 11359840

[36] Schwabe RF Bataller R Brenner DA. Human Hepatic Stellate Cells Express CCR5 and RANTES to Induce Proliferation and Migration. Am J Physiol Gastrointest Liver Physiol (2003) 285(5):G949–58. doi: 10.1152/ajpgi.00215.2003 12829440

[37] Hong F Tuyama A Lee TF Loke J Agarwal R Cheng X. Hepatic Stellate Cells Express Functional CXCR4: Role in Stromal Cell-Derived Factor-1alpha-Mediated Stellate Cell Activation. Hepatology (2009) 49(6):2055–67. doi: 10.1002/hep.22890 PMC2893547 19434726

[38] Hellerbrand Wang SC Tsukamoto H Brenner DA Rippe RA. Expression of Intracellular Adhesion Molecule 1 by Activated Hepatic Stellate Cells. Hepatology (1996) 24(3):670–6. doi: 10.1002/hep.510240333 8781341

[39] Fujita T Soontrapa K Ito Y Iwaisako K Moniaga CS Asagiri M. Hepatic Stellate Cells Relay Inflammation Signaling From Sinusoids to Parenchyma in Mouse Models of Immune-Mediated Hepatitis. Hepatology (2016) 63(4):1325–39. doi: 10.1002/hep.28112 26248612

[40] Vinas O Bataller R Sancho-Bru P Gines P Berenguer C Enrich C. Human Hepatic Stellate Cells Show Features of Antigen-Presenting Cells and Stimulate Lymphocyte Proliferation. Hepatology (2003) 38(4):919–29. doi: 10.1002/hep.1840380418 14512879

[41] Winau F Hegasy G Weiskirchen R Weber S Cassan C Sieling PA. Ito Cells Are Liver-Resident Antigen-Presenting Cells for Activating T Cell Responses. Immunity (2007) 26(1):117–29. doi: 10.1016/j.immuni.2006.11.011 17239632

[42] Horst AK Neumann K Diehl L Tiegs G. Modulation of Liver Tolerance by Conventional and Nonconventional Antigen-Presenting Cells and Regulatory Immune Cells. Cell Mol Immunol (2016) 13(3):277–92. doi: 10.1038/cmi.2015.112 PMC4856800 27041638

[43] Ichikawa S Mucida D Tyznik AJ Kronenberg M Cheroutre H. Hepatic Stellate Cells Function as Regulatory Bystanders. J Immunol (2011) 186(10):5549–55. doi: 10.4049/jimmunol.1003917 PMC3132534 21460203

[44] Sumpter TL Dangi A Matta BM Huang C Stolz DB Vodovotz Y. Hepatic Stellate Cells Undermine the Allostimulatory Function of Liver Myeloid Dendritic Cells via STAT3-Dependent Induction of IDO. J Immunol (2012) 189(8):3848–58. doi: 10.4049/jimmunol.1200819 PMC3466356 22962681

[45] Schildberg FA Wojtalla A Siegmund SV Endl E Diehl L Abdullah Z. Murine Hepatic Stellate Cells Veto CD8 T Cell Activation by a CD54-Dependent Mechanism. Hepatology (2011) 54(1):262–72. doi: 10.1002/hep.24352 21488077

[46] Yu MC Chen CH Liang X Wang L Gandhi CR Fung JJ. Inhibition of T-Cell Responses by Hepatic Stellate Cells via B7-H1-Mediated T-Cell Apoptosis in Mice. Hepatology (2004) 40(6):1312–21. doi: 10.1002/hep.20488 15565659

[47] Chen CH Kuo LM Chang Y Wu W Goldbach C Ross MA. In Vivo Immune Modulatory Activity of Hepatic Stellate Cells in Mice. Hepatology (2006) 44(5):1171–81. doi: 10.1002/hep.21379 17058227

[48] Charles R Chou HS Wang L Fung JJ Lu L Qian S. Human Hepatic Stellate Cells Inhibit T-Cell Response Through B7-H1 Pathway. Transplantation (2013) 96(1):17–24. doi: 10.1097/TP.0b013e318294caae 23756770 PMC3696433

[49] Li Y Lu L Qian SG Fung JJ Lin F. Hepatic Stellate Cells Directly Inhibit B Cells via Programmed Death-Ligand 1. J Immunol (2016) 196(4):1617–25. doi: 10.4049/jimmunol.1501737 PMC4784705 26755818

[50] Jiang G Yang HR Wang L Wildey GM Fung J Qian S. Hepatic Stellate Cells Preferentially Expand Allogeneic CD4+ CD25+ FoxP3+ Regulatory T Cells in an IL-2-Dependent Manner. Transplantation (2008) 86(11):1492–502. doi: 10.1097/TP.0b013e31818bfd13 PMC2888269 19077880

[51] Dunham RM Thapa M Velazquez VM Elrod EJ Denning TL Pulendran B. Hepatic Stellate Cells Preferentially Induce Foxp3+ Regulatory T Cells by Production of Retinoic Acid. J Immunol (2013) 190(5):2009–16. doi: 10.4049/jimmunol.1201937 PMC3575565 23359509

[52] Chou HS Hsieh CC Yang HR Wang L Arakawa Y Brown K. Hepatic Stellate Cells Regulate Immune Response by Way of Induction of Myeloid Suppressor Cells in Mice. Hepatology (2011) 53(3):1007–19. doi: 10.1002/hep.24162 PMC3079329 21374665

[53] Puche JE Lee YA Jiao J Aloman C Fiel MI Munoz U. A Novel Murine Model to Deplete Hepatic Stellate Cells Uncovers Their Role in Amplifying Liver Damage in Mice. Hepatology (2013) 57(1):339–50. doi: 10.1002/hep.26053 PMC3522764 22961591

[54] Wang ZY Keogh A Waldt A Cuttat R Neri M Zhu S. Single-Cell and Bulk Transcriptomics of the Liver Reveals Potential Targets of NASH With Fibrosis. Sci Rep (2021) 11(1):19396. doi: 10.1038/s41598-021-98806-y 34588551 PMC8481490

[55] Matsuda M Seki E. Hepatic Stellate Cell-Macrophage Crosstalk in Liver Fibrosis and Carcinogenesis. Semin Liver Dis (2020) 3:307–20. doi: 10.1055/s-0040-1708876 PMC7484001 32242330

[56] Ramachandran P Dobie R Wilson-Kanamori JR Dora EF Henderson BEP Luu NT. Resolving the Fibrotic Niche of Human Liver Cirrhosis at Single-Cell Level. Nature (2019) 575(7783):512–8. doi: 10.1038/s41586-019-1631-3 PMC6876711 31597160

[57] Chang J Hisamatsu T Shimamura K Yoneno K Adachi M Naruse H. Activated Hepatic Stellate Cells Mediate the Differentiation of Macrophages. Hepatol Res (2013) 43(6):658–69. doi: 10.1111/j.1872-034X.2012.01111.x 23107150

[58] Aoyama T Inokuchi S Brenner DA Seki E. CX3CL1-CX3CR1 Interaction Prevents Carbon Tetrachloride-Induced Liver Inflammation and Fibrosis in Mice. Hepatology (2010) 52(4):1390–400. doi: 10.1002/hep.23795 PMC2947579 20683935

[59] Karlmark KR Zimmermann HW Roderburg C Gassler N Wasmuth HE Luedde T. The Fractalkine Receptor CX_3_CR1 Protects Against Liver Fibrosis by Controlling Differentiation and Survival of Infiltrating Hepatic Monocytes. Hepatology (2010) 5:1769–82. doi: 10.1002/hep.23894 21038415

[60] Thomas JA Pope C Wojtacha D Robson AJ Gordon-Walker TT Hartland S. Macrophage Therapy for Murine Liver Fibrosis Recruits Host Effector Cells Improving Fibrosis, Regeneration, and Function. Hepatology (2011) 53(6):2003–15. doi: 10.1002/hep.24315 21433043

[61] Ramachandran P Pellicoro A Vernon MA Boulter L Aucott RL Ali A. Differential Ly-6C Expression Identifies the Recruited Macrophage Phenotype, Which Orchestrates the Regression of Murine Liver Fibrosis. Proc Natl Acad Sci USA (2012) 109(46):E3186–95. doi: 10.1073/pnas.1119964109 PMC3503234 23100531

[62] Kaufmann B Reca A Kim AD Feldstein AE. Novel Mechanisms for Resolution of Liver Inflammation: Therapeutic Implications. Semin Liver Dis (2021) 2:150–62. doi: 10.1055/s-0041-1723031 34107544

[63] Zhou X Franklin RA Adler M Jacox JB Bailis W Shyer JA. Circuit Design Features of a Stable Two-Cell System. Cell (2018) 172(4):744–57.e17. doi: 10.1016/j.cell.2018.01.015 29398113 PMC7377352

[64] Adler M Mayo A Zhou X Franklin RA Meizlish ML Medzhitov R. Principles of Cell Circuits for Tissue Repair and Fibrosis. iScience (2020) 23(2):100841. doi: 10.1016/j.isci.2020.100841 32058955 PMC7005469

[65] Safadi R Ohta M Alvarez CE Fiel MI Bansal M Mehal WZ. Immune Stimulation of Hepatic Fibrogenesis by CD8 Cells and Attenuation by Transgenic Interleukin-10 From Hepatocytes. Gastroenterology (2004) 127(3):870–82. doi: 10.1053/j.gastro.2004.04.062 15362042

[66] Thapa M Chinnadurai R Velazquez VM Tedesco D Elrod E Han JH. Liver Fibrosis Occurs Through Dysregulation of MyD88-Dependent Innate B-Cell Activity. Hepatology (2015) 61(6):2067–79. doi: 10.1002/hep.27761 PMC4441566 25711908

[67] Trasino SE Tang XH Jessurun J Gudas LJ. A Retinoic Acid Receptor β2 Agonist Reduces Hepatic Stellate Cell Activation in Nonalcoholic Fatty Liver Disease. J Mol Med (Berl) (2016) 94(10):1143–51. doi: 10.1007/s00109-016-1434-z PMC5053866 27271256

[68] Kartasheva-Ebertz DM Pol S Lagaye S. Retinoic Acid: A New Old Friend of IL-17A in the Immune Pathogeny of Liver Fibrosis. Front Immunol (2021) 12. doi: 10.3389/fimmu.2021.691073 PMC8239722 34211477

[69] Jimenez Calvente C Tameda M Johnson CD Del Pilar H Chin Lin Y Andronikou N. Neutrophils Contribute to Spontaneous Resolution of Liver Inflammation and Fibrosis via microRNA-223. J Clin Invest (2019) 130. doi: 10.1172/JCI122258 PMC6763256 31295147

[70] Rockey DC Maher JJ Jarnagin WR Gabbiani G Friedman SL. Inhibition of Rat Hepatic Lipocyte Activation in Culture by Interferon- Gamma. Hepatology (1992) 16(3):776–84. doi: 10.1002/hep.1840160325 1505921

[71] Wynn TA. Fibrotic Disease and the T(H)1/T(H)2 Paradigm. Nat Rev Immunol (2004) 4(8):583–94. doi: 10.1038/nri1412 PMC2702150 15286725

[72] Weng H Mertens PR Gressner AM Dooley S. IFN-Gamma Abrogates Profibrogenic TGF-Beta Signaling in Liver by Targeting Expression of Inhibitory and Receptor Smads. J Hepatol (2007) 46(2):295–303. doi: 10.1016/j.jhep.2006.09.014 17125875

[73] Ramachandran P Matchett KP Dobie R Wilson-Kanamori JR Henderson NC. Single-Cell Technologies in Hepatology: New Insights Into Liver Biology and Disease Pathogenesis. Nat Rev Gastroenterol Hepatol (2020) 17(8):457–72. doi: 10.1038/s41575-020-0304-x 32483353

[74] Arrese M Cabrera D Kalergis AM Feldstein AE. Innate Immunity and Inflammation in NAFLD/NASH. Digestive Dis Sci (2016) 5:1294–1303. doi: 10.1007/s10620-016-4049-x PMC4948286 26841783

[75] Inzaugarat ME Johnson CD Holtmann TM McGeough MD Trautwein C Papouchado BG. NLR Family Pyrin Domain-Containing 3 Inflammasome Activation in Hepatic Stellate Cells Induces Liver Fibrosis in Mice. Hepatology (2019). doi: 10.1002/hep.30252 PMC6351190 30180270

[76] Gaul S Leszczynska A Alegre F Kaufmann B Johnson CD Adams LA. Hepatocyte Pyroptosis and Release of Inflammasome Particles Induce Stellate Cell Activation and Liver Fibrosis. J Hepatol (2020) 1:156–167. 10.1016/j.jhep.2020.07.041 PMC7749849 32763266

[77] Mridha AR Wree A Robertson AAB Yeh MM Johnson CD Van Rooyen DM. NLRP3 Inflammasome Blockade Reduces Liver Inflammation and Fibrosis in Experimental NASH in Mice. J Hepatol (2017) 66(5):1037–46. doi: 10.1016/j.jhep.2017.01.022 PMC6536116 28167322

[78] Chu H Williams B Schnabl B. Gut Microbiota, Fatty Liver Disease, and Hepatocellular Carcinoma. Liver Res (2018) 2(1):43–51. doi: 10.1016/j.livres.2017.11.005 30416839 PMC6223644

[79] Loomba R Seguritan V Li W Long T Klitgord N Bhatt A. Gut Microbiome-Based Metagenomic Signature for Non-Invasive Detection of Advanced Fibrosis in Human Nonalcoholic Fatty Liver Disease. Cell Metab (2017) 25(5):1054–62.e5. doi: 10.1016/j.cmet.2017.04.001 28467925 PMC5502730

[80] Caussy C Tripathi A Humphrey G Bassirian S Singh S Faulkner C. A Gut Microbiome Signature for Cirrhosis Due to Nonalcoholic Fatty Liver Disease. Nat Commun (2019) 10(1):1406. doi: 10.1038/s41467-019-09455-9 30926798 PMC6440960

[81] Yuan J Chen C Cui J Lu J Yan C Wei X. Fatty Liver Disease Caused by High-Alcohol-Producing Klebsiella Pneumoniae. Cell Metab (2019) 30(4):675–88.e7. doi: 10.1016/j.cmet.2019.08.018 31543403

[82] Carter JK Bhattacharya D Borgerding JN Fiel MI Faith JJ Friedman SL. Modeling Dysbiosis of Human NASH in Mice: Loss of Gut Microbiome Diversity and Overgrowth of Erysipelotrichales. PloS One (2021) 16(1):e0244763. doi: 10.1371/journal.pone.0244763 33395434 PMC7781477

[83] Schuppan D Surabattula R Wang XY. Determinants of Fibrosis Progression and Regression in NASH. J Hepatol (2018) 68(2):238–50. doi: 10.1016/j.jhep.2017.11.012 29154966

[84] Kazankov K Jorgensen SMD Thomsen KL Moller HJ Vilstrup H George J. The Role of Macrophages in Nonalcoholic Fatty Liver Disease and Nonalcoholic Steatohepatitis. Nat Rev Gastroenterol Hepatol (2019) 16(3):145–59. doi: 10.1038/s41575-018-0082-x 30482910

[85] Luo X Li H Ma L Zhou J Guo X Woo SL. Expression of STING Is Increased in Liver Tissues From Patients With NAFLD and Promotes Macrophage-Mediated Hepatic Inflammation and Fibrosis in Mice. Gastroenterology (2018) 155(6):1971–84.e4. doi: 10.1053/j.gastro.2018.09.010 30213555 PMC6279491

[86] Yu Y Liu Y An W Song J Zhang Y Zhao X. STING-Mediated Inflammation in Kupffer Cells Contributes to Progression of Nonalcoholic Steatohepatitis. J Clin Invest (2019) 2:546–55. 10.1172/JCI121842 PMC6355218 30561388

[87] Stienstra R Saudale F Duval C Keshtkar S Groener JEM van Rooijen N. Kupffer Cells Promote Hepatic Steatosis via Interleukin-1beta-Dependent Suppression of Peroxisome Proliferator-Activated Receptor Alpha Activity. Hepatology (2010) 2:511–22. doi: 10.1002/hep.23337 20054868

[88] Huang W Metlakunta A Dedousis N Zhang P Sipula I Dube JJ. Depletion of Liver Kupffer Cells Prevents the Development of Diet-Induced Hepatic Steatosis and Insulin Resistance. Diabetes (2010) 2:347–357. doi: 10.2337/db09-0016 PMC2809951 19934001

[89] Svendsen P Graversen JH Etzerodt A Hager H Røge R Grønbæk H. Antibody-Directed Glucocorticoid Targeting to CD163 in M2-Type Macrophages Attenuates Fructose-Induced Liver Inflammatory Changes. Mol Ther Methods Clin Dev (2016) 4:50–61. doi: 10.1016/j.omtm.2016.11.004 28344991 PMC5363319

[90] Baeck C Wehr A Karlmark KR Heymann F Vucur M Gassler N. Pharmacological Inhibition of the Chemokine CCL2 (MCP-1) Diminishes Liver Macrophage Infiltration and Steatohepatitis in Chronic Hepatic Injury. Gut (2011) 3:416–426. doi: 10.1136/gutjnl-2011-300304 21813474

[91] Krenkel O Puengel T Govaere O Abdallah AT Mossanen JC Kohlhepp M. Therapeutic Inhibition of Inflammatory Monocyte Recruitment Reduces Steatohepatitis and Liver Fibrosis. Hepatology (2018) 67(4):1270–83. doi: 10.1002/hep.29544 28940700

[92] Lefere S Puengel T Hundertmark J Penners C Frank AK Guillot A. Differential Effects of Selective- and Pan-PPAR Agonists on Experimental Steatohepatitis and Hepatic Macrophages(☆). J Hepatol (2020) 4:757–770. doi: 10.1016/j.jhep.2020.04.025 32360434

[93] Hou J Zhang J Cui P Zhou Y Liu C Wu X. TREM2 Sustains Macrophage-Hepatocyte Metabolic Coordination in Nonalcoholic Fatty Liver Disease and Sepsis. J Clin Invest (2021) 131(4). doi: 10.1172/JCI135197 PMC7880419 33586673

[94] Coelho I Duarte N Barros A Macedo MP Penha-Gonçalves C. Trem-2 Promotes Emergence of Restorative Macrophages and Endothelial Cells During Recovery From Hepatic Tissue Damage. Front Immunol (2021). doi: 10.3389/fimmu.2020.616044 PMC7897679 33628208

[95] Tran S Baba I Poupel L Dussaud S Moreau M Gélineau A. Impaired Kupffer Cell Self-Renewal Alters the Liver Response to Lipid Overload During Non-Alcoholic Steatohepatitis. Immunity (2020) 3:627–640. doi: 10.1016/j.immuni.2020.06.003 32562600

[96] Bonnardel J T'Jonck W Gaublomme D Browaeys R Scott CL Martens L. Stellate Cells, Hepatocytes, and Endothelial Cells Imprint the Kupffer Cell Identity on Monocytes Colonizing the Liver Macrophage Niche. Immunity (2019) 4:638–54. doi: 10.1016/j.immuni.2019.08.017 PMC6876284 31561945

[97] Itoh M Kato H Suganami T Konuma K Marumoto Y Terai S. Hepatic Crown-Like Structure: A Unique Histological Feature in non-Alcoholic Steatohepatitis in Mice and Humans. PloS One (2013) 12:e82163. doi: 10.1371/journal.pone.0082163 PMC3859576 24349208

[98] Itoh M Suganami T Kato H Kanai S Shirakawa I Sakai T. CD11c+ Resident Macrophages Drive Hepatocyte Death-Triggered Liver Fibrosis in a Murine Model of Nonalcoholic Steatohepatitis. JCI Insight (2017), e92902. doi: 10.1172/jci.insight.92902 PMC5752377 29202448

[99] Daemen S Gainullina A Kalugotla G He L Chan MM Beals JW. Dynamic Shifts in the Composition of Resident and Recruited Macrophages Influence Tissue Remodeling in NASH. Cell Rep (2021) 2:108626. doi: 10.1016/j.celrep.2020.108626 PMC7877246 33440159

[100] Cai B Dongiovanni P Corey KE Wang X Shmarakov IO Zheng Z. Macrophage MerTK Promotes Liver Fibrosis in Nonalcoholic Steatohepatitis. Cell Metab (2020) 31(2):406–21.e7. doi: 10.1016/j.cmet.2019.11.013 31839486 PMC7004886

[101] Dalli J Zhu M Vlasenko NA Deng B Haeggström JZ Petasis NA. The Novel 13S,14S-Epoxy-Maresin Is Converted by Human Macrophages to Maresin 1 (MaR1), Inhibits Leukotriene A4 Hydrolase (LTA4H), and Shifts Macrophage Phenotype. FASEB J (2013) 7:2573–83. doi: 10.1096/fj.13-227728 PMC3688739 23504711

[102] Han YH Shin KO Kim JY Khadka DB Kim HJ Lee YM. A Maresin 1/Rorα/12-Lipoxygenase Autoregulatory Circuit Prevents Inflammation and Progression of Nonalcoholic Steatohepatitis. J Clin Invest (2019) 4:1684–1698. doi: 10.1172/JCI124219 PMC6436872 30855276

[103] Puchalska P Martin SE Huang X Lengfeld JE Daniel B Graham MJ. Hepatocyte-Macrophage Acetoacetate Shuttle Protects Against Tissue Fibrosis. Cell Metab (2019) 29(2):383–98.e387. doi: 10.1016/j.cmet.2018.10.015 30449686 PMC6559243

[104] Hwang S Yun H Moon S Cho YE Gao B. Role of Neutrophils in the Pathogenesis of Nonalcoholic Steatohepatitis. Front Endocrinol (Lausanne) (2021) 12. doi: 10.3389/fendo.2021.751802 PMC8542869 34707573

[105] Pulli B Ali M Iwamoto Y Zeller MW Schob S Linnoila JJ. Myeloperoxidase-Hepatocyte-Stellate Cell Cross Talk Promotes Hepatocyte Injury and Fibrosis in Experimental Nonalcoholic Steatohepatitis. Antioxid Redox Signal (2015) 16:1255–69. doi: 10.1089/ars.2014.6108 PMC4677570 26058518

[106] Koop AC Thiele ND Steins D Michaëlsson E Wehmeyer M Scheja L. Therapeutic Targeting of Myeloperoxidase Attenuates NASH in Mice. Hepatol Commun (2020) 10:1441–1458. doi: 10.1002/hep4.1566 PMC7527691 33024915

[107] Zhou Z Xu MJ Cai Y Wang W Jiang JX Varga ZV. Neutrophil-Hepatic Stellate Cell Interactions Promote Fibrosis in Experimental Steatohepatitis. Cell Mol Gastroenterol Hepatol (2018) 3:399–413. doi: 10.1016/j.jcmgh.2018.01.003 PMC5852390 29552626

[108] Radaeva S Sun R Jaruga B Nguyen VT Tian Z Gao B. Natural Killer Cells Ameliorate Liver Fibrosis by Killing Activated Stellate Cells in NKG2D-Dependent and Tumor Necrosis Factor-Related Apoptosis-Inducing Ligand-Dependent Manners. Gastroenterol (2006) 2:435–52. doi: 10.1053/j.gastro.2005.10.055 16472598

[109] Melhem A Muhanna N Bishara A Alvarez CE Ilan Y Bishara T. Anti-Fibrotic Activity of NK Cells in Experimental Liver Injury Through Killing of Activated HSC. J Hepatol (2006) 45(1):60–71. doi: 10.1016/j.jhep.2005.12.025 16515819

[110] Radaeva S Wang L Radaev S Jeong WI Park O Gao B. Retinoic Acid Signaling Sensitizes Hepatic Stellate Cells to NK Cell Killing via Upregulation of NK Cell Activating Ligand RAE1. Am J Physiol Gastrointest Liver Physiol (2007) 293(4):G809–16. doi: 10.1152/ajpgi.00212.2007 17673545

[111] Gur C Doron S Kfir-Erenfeld S Horwitz E Abu-Tair L Safadi R. NKp46-Mediated Killing of Human and Mouse Hepatic Stellate Cells Attenuates Liver Fibrosis. Gut (2011) 6:885–93. doi: 10.1136/gutjnl-2011-301400 22198715

[112] Martínez-Chantar ML Delgado TC Beraza N. Revisiting the Role of Natural Killer Cells in Non-Alcoholic Fatty Liver Disease. Front Immunol (2021) 12. doi: 10.3389/fimmu.2021.640869 PMC7930075 33679803

[113] Amer J Salhab A Noureddin M Doron S Abu-Tair L Ghantous R. Insulin Signaling as a Potential Natural Killer Cell Checkpoint in Fatty Liver Disease. Hepatol Commun (2018) 3:285–98. doi: 10.1002/hep4.1146 PMC5831020 29507903

[114] Cuff AO Sillito F Dertschnig S Hall A Luong TV Chakraverty R. The Obese Liver Environment Mediates Conversion of NK Cells to a Less Cytotoxic ILC1-Like Phenotype. Front Immunol (2019) 10. doi: 10.1101/576538 PMC6749082 31572388

[115] Maricic I Marrero I Eguchi A Nakamura R Johnson CD Dasgupta S. Differential Activation of Hepatic Invariant NKT Cell Subsets Plays a Key Role in Progression of Nonalcoholic Steatohepatitis. J Immunol (2018) 201(10):3017–35. doi: 10.4049/jimmunol.1800614 PMC6219905 30322964

[116] Sutti S Albano EA-O. Adaptive Immunity: An Emerging Player in the Progression of NAFLD. Nat Rev Gastroenterol Hepatol (2020) 2:81–92. doi: 10.1038/s41575-019-0210-2 PMC7222953 31605031

[117] Sicklick JK Li YX Choi SS Qi Y Chen W Bustamante M. Role for Hedgehog Signaling in Hepatic Stellate Cell Activation and Viability. Lab Invest (2005) 85(11):1368–80. doi: 10.1038/labinvest.3700349 16170335

[118] Syn WK Agboola KM Swiderska M Michelotti GA Liaskou E Pang H. NKT-Associated Hedgehog and Osteopontin Drive Fibrogenesis in Non-Alcoholic Fatty Liver Disease. Gut (2012) 61(9):1323–9. doi: 10.1136/gutjnl-2011-301857 PMC3578424 22427237

[119] Arriazu E Ge X Leung TM Magdaleno F Lopategi A Lu Y. Signalling via the Osteopontin and High Mobility Group Box-1 Axis Drives the Fibrogenic Response to Liver Injury. Gut (2017) 6:1123–37. doi: 10.1136/gutjnl-2015-310752 PMC5532463 26818617

[120] Choi SS Omenetti A Witek RP Moylan CA Syn W-K Jung Y. Hedgehog Pathway Activation and Epithelial-to-Mesenchymal Transitions During Myofibroblastic Transformation of Rat Hepatic Cells in Culture and Cirrhosis. Am J Physiol Gastrointest Liver Physiol (2009) 6:G1093–106. doi: 10.1152/ajpgi.00292.2009 PMC2850083 19815628

[121] Wolf MJ Adili A Piotrowitz K Abdullah Z Boege Y Stemmer K. Metabolic Activation of Intrahepatic CD8+ T Cells and NKT Cells Causes Nonalcoholic Steatohepatitis and Liver Cancer via Cross-Talk With Hepatocytes. Cancer Cell (2014) 4:549–64. doi: 10.1016/j.ccell.2014.09.003 25314080

[122] Park O Jeong WI Wang L Wang H Lian ZX Gershwin ME. Diverse Roles of Invariant Natural Killer T Cells in Liver Injury and Fibrosis Induced by Carbon Tetrachloride. Hepatology (2009) 49(5):1683–94. doi: 10.1002/hep.22813 PMC2772879 19205035

[123] Mitra A Satelli A Yan J Xueqing X Gagea M Hunter CA. IL-30 (IL27p28) Attenuates Liver Fibrosis Through Inducing NKG2D-Rae1 Interaction Between NKT and Activated Hepatic Stellate Cells in Mice. Hepatology (2014) 6:2027–39. doi: 10.1002/hep.27392 PMC4245364 25351459

[124] Hunter S Willcox CR Davey MS Kasatskaya SA Jeffery HC Chudakov DM. Human Liver Infiltrating γδ T Cells are Composed of Clonally Expanded Circulating and Tissue-Resident Populations. J Hepatol (2018). doi: 10.1016/j.jhep.2018.05.007 PMC6089840 29758330

[125] Hammerich L Bangen JM Govaere O Zimmermann HW Gassler N Huss S. Chemokine Receptor CCR6-Dependent Accumulation of Gammadelta T-Cells in Injured Liver Restricts Hepatic Inflammation and Fibrosis. Hepatology (2013) 2:630–642. doi: 10.1002/hep.26697 PMC4139146 23959575

[126] Liu M Hu Y Yuan Y Tian Z Zhang C. γδt Cells Suppress Liver Fibrosis via Strong Cytolysis and Enhanced NK Cell-Mediated Cytotoxicity Against Hepatic Stellate Cells. Front Immunol (2019) 10. doi: 10.3389/fimmu.2019.00477 PMC6428727 30930903

[127] Hegde P Weiss E Paradis V Wan J Mabire M Sukriti S. Mucosal-Associated Invariant T Cells are a Profibrogenic Immune Cell Population in the Liver. Nat Commun (2018) 9(1):2146. doi: 10.1038/s41467-018-04450-y 29858567 PMC5984626

[128] Böttcher K Rombouts K Saffioti F Roccarina D Rosselli M Hall A. MAIT Cells Are Chronically Activated in Patients With Autoimmune Liver Disease and Promote Profibrogenic Hepatic Stellate Cell Activation. Hepatology (2018) 1:172–86. doi: 10.1002/hep.29782 29328499

[129] Yeh MM Brunt EM. Pathological Features of Fatty Liver Disease. Gastroenterol (2014) 4:754–764. doi: 10.1053/j.gastro.2014.07.056 25109884

[130] Hirsova P Bamidele AO Wang H Povero D Revelo XS. Emerging Roles of T Cells in the Pathogenesis of Nonalcoholic Steatohepatitis and Hepatocellular Carcinoma. Front Endocrinol (Lausanne) (2021) 12. doi: 10.3389/fendo.2021.760860 PMC8581300 34777255

[131] Inzaugarat ME Ferreya Solari NE Billordo LA Abecasis R Gadano AC Cherñavsky AC. Altered Phenotype and Functionality of Circulating Immune Cells Characterize Adult Patients With Nonalcoholic Steatohepatitis. J Clin Immunol (2011) 6:1120–30. doi: 10.1007/s10875-011-9571-1 21845516

[132] Ghazarian M Revelo XS Nøhr MK Luck H Zeng K Lei H. Type I Interferon Responses Drive Intrahepatic T Cells to Promote Metabolic Syndrome. Sci Immunol (2017) 10:eaai7616. doi: 10.1126/sciimmunol.aai7616 PMC5447456 28567448

[133] Bhattacharjee J Kirby M Softic S Miles L Salazar-Gonzalez RM Shivakumar P. Hepatic Natural Killer T-Cell and CD8+ T-Cell Signatures in Mice With Nonalcoholic Steatohepatitis. Hepatol Commun (2017) 4:299–310. doi: 10.1002/hep4.1041 PMC5687094 29152605

[134] Grohmann M Wiede F Dodd GT Gurzov EN Ooi GJ Butt T. Obesity Drives STAT-1-Dependent NASH and STAT-3-Dependent HCC. Cell (2018) 175(5):1289–1306.e1220. doi: 10.1016/j.cell.2018.09.053 30454647 PMC6242467

[135] Haas JT Vonghia L Mogilenko DA Verrijken A Molendi-Coste O Fleury S. Transcriptional Network Analysis Implicates Altered Hepatic Immune Function in NASH Development and Resolution. Nat Metab (2019) 6:604–614. doi: 10.1038/s42255-019-0076-1 PMC6837876 31701087

[136] Dudek M Pfister D Donakonda S Filpe P Schneider A Laschinger M. Auto-Aggressive CXCR6(+) CD8 T Cells Cause Liver Immune Pathology in NASH. Nature (2021) 7854:444–9. doi: 10.1055/s-0041-1740803 33762736

[137] Luo XY Takahara T Kawai K Fujino M Sugiyama T Tsuneyama K. IFN-γ Deficiency Attenuates Hepatic Inflammation and Fibrosis in a Steatohepatitis Model Induced by a Methionine- and Choline-Deficient High-Fat Diet. Gastroenterol (2013) 12:G891–9. doi: 10.1152/ajpgi.00193.2013 24136786

[138] Neumann K Kruse N Szilagyi B Erben U Rudolph C Flach A. Connecting Liver and Gut: Murine Liver Sinusoidal Endothelium Induces Gut Tropism of CD4+ T Cells via Retinoic Acid. Hepatology (2012) 6:1976–84. doi: 10.1002/hep.24816 22109893

[139] Rai RP Liu Y Iyer SS Liu S Gupta B Desai C. Blocking Integrin Alpha4beta7-Mediated CD4 T Cell Recruitment to the Intestine and Liver Protects Mice From Western Diet-Induced non-Alcoholic Steatohepatitis. J Hepatol (2020) 73(5):1013–22. doi: 10.1016/j.jhep.2020.05.047 PMC7839272 32540177

[140] Bruzzì S Sutti S Giudici G Burlone ME Ramavath NN Toscani A. B2-Lymphocyte Responses to Oxidative Stress-Derived Antigens Contribute to the Evolution of Nonalcoholic Fatty Liver Disease (NAFLD). Free Radic Biol Med (2018) 124:249–259. doi: 10.1016/j.freeradbiomed.2018.06.015 29920340

[141] Barrow F Khan S Wang H Revelo XA-OX. The Emerging Role of B Cells in the Pathogenesis of NAFLD. Hepatology (2021) 4:2277–86. doi: 10.1002/hep.31889 PMC8463421 33961302

[142] Miyake T Abe M Tokumoto Y Hirooka M Furukawa S Kumagi T. B Cell-Activating Factor is Associated With the Histological Severity of Nonalcoholic Fatty Liver Disease. Hepatol Int (2013) 2:539–547. 10.1007/s12072-012-9345-8 26201785

[143] McPherson S Henderson E Burt AD Day CP Anstee QM. Serum Immunoglobulin Levels Predict Fibrosis in Patients With Non-Alcoholic Fatty Liver Disease. J Hepatol (2014) 5:1055–62. doi: 10.1016/j.jhep.2014.01.010 24445215

[144] Zhang F Jiang WW Li X Qiu XY Wu Z Chi YJ. Role of Intrahepatic B Cells in Non-Alcoholic Fatty Liver Disease by Secreting Pro-Inflammatory Cytokines and Regulating Intrahepatic T Cells. J Dig Dis (2016) 7:464–74. doi: 10.1111/1751-2980.12362 27216040

[145] Barrow F Khan S Fredrickson G Wang H Dietsche K Parthiban P. Microbiota-Driven Activation of Intrahepatic B Cells Aggravates NASH Through Innate and Adaptive Signaling. Hepatology (2021) 2:704–22. doi: 10.1002/hep.31755 PMC8377092 33609303

[146] Wang ZY Keogh A Waldt A Cuttat R Neri M Zhu S. Single-Cell and Bulk Transcriptomics of the Liver Reveals Potential Targets of NASH With Fibrosis. Sci Rep (2021) 1:19396. doi: 10.1038/s41598-021-98806-y PMC8481490 34588551

[147] Saviano A Henderson NC Baumert TF. Single-Cell Genomics and Spatial Transcriptomics: Discovery of Novel Cell States and Cellular Interactions in Liver Physiology and Disease Biology. J Hepatol (2020) 73(5):1219–30. doi: 10.1016/j.jhep.2020.06.004 PMC7116221 32534107

[148] Rosenthal SB Liu X Ganguly S Dhar D Pasillas MP Ricciardelli E. Heterogeneity of Hepatic Stellate Cells in a Mouse Model of non-Alcoholic Steatohepatitis (NASH). Hepatology (2021) 2:667–85. 10.1002/hep.31743 PMC8346581 33550587

[149] Amor C Feucht J Leibold J Ho YJ Zhu C Alonso-Curbelo D. Senolytic CAR T Cells Reverse Senescence-Associated Pathologies. Nature (2020) 583(7814):127–32. doi: 10.1038/s41586-020-2403-9 PMC7583560 32555459

[150] Li F Huangyang P Burrows M Guo K Riscal R Godfrey J. FBP1 Loss Disrupts Liver Metabolism and Promotes Tumorigenesis Through a Hepatic Stellate Cell Senescence Secretome. Nat Cell Biol (2020) 22(6):728–39. doi: 10.1038/s41556-020-0511-2 PMC7286794 32367049

[151] Efremova M Vento-Tormo M Teichmann S Vento-Tormo R. CellPhoneDB: Inferring Cell-Cell Communication From Combined Expression of Multi-Subunit Ligand-Receptor Complexes. Nat Protoc (2020) 4:1484–1506. doi: 10.1101/680926 32103204

[152] Armingol E Officer A Harismendy O Lewis NE. Deciphering Cell-Cell Interactions and Communication From Gene Expression. Nat Rev Genet (2020) 2:71–88. doi: 10.1038/s41576-020-00292-x PMC7649713 33168968

